# Evaluation of a Gel Containing a *Propionibacterium* Extract in an In Vivo Model of Wound Healing

**DOI:** 10.3390/ijms23094708

**Published:** 2022-04-24

**Authors:** Campolo M., Gallo G., Roviello F., Ardizzone A., La Torre M., Filippone A., Lanza M., Cuzzocrea S., Siroli L., Esposito E.

**Affiliations:** 1 Department of Chemical, Biological, Pharmaceutical and Environmental Sciences, University of Messina, Viale Ferdinando Stagno D’Alcontres, 31-98166 Messina, Italy; michela.campolo@unime.it (C.M.); aleardizzone@unime.it (A.A.); afilippone@unime.it (F.A.); mlanza@unime.it (L.M.); salvator@unime.it (C.S.); emanuela.esposito@unime.it (E.E.); 2 Unit of General Surgery and Surgical Oncology, Department of Medicine, Surgery and Neurosciences, University of Siena, 53100 Siena, Italy; franco.roviello@unisi.it; 3 Coloproctology Unit, Salvator Mundi International Hospital, UPMC University of Pittsburgh Medical Center, 00152 Rome, Italy; dott.marcolatorre@gmail.com; 4 Department of Agricultural and Food Sciences, Campus of Food Science, Piazza Goidanich 60, 47521 Cesena, Province of Forlì-Cesena, Italy; lorenzo.siroli2@unibo.it

**Keywords:** Emorsan^®^ Gel, *Propionibacterium* extract, bacterial lysate, wound healing, topical treatment, skin care, antioxidant effect, anti-inflammatory effect

## Abstract

Inappropriate wound healing (WH) management can cause significant comorbidities, especially in patients affected by chronic and metabolic diseases, such as diabetes. WH involves several different, partially overlapping processes, including hemostasis, inflammation, cell proliferation, and remodeling. Oxidative stress in WH contributes to WH impairment because of the overexpression of radical oxygen species (ROS) and nitrogen species (RNS). This study aimed to evaluate the in vitro antioxidative action of a gel containing a *Propionibacterium* extract (Emorsan^®^ Gel) and assess its skin re-epithelialization properties in a mouse model of WH. The scavenging effects of the bacterial extract were assessed in vitro through the ABTS and DPPH assays and in L-929 murine fibroblasts. The effects of the Emorsan^®^ Gel were studied in vivo in a murine model of WH. After WH induction, mice were treated daily with vehicle or Emorsan^®^ Gel for 6 or 12 days. According to the in vitro tests, the *Propionibacterium* extract exerted an inhibitory effect on ROS and RNS, consequently leading to the reduction in malondialdehyde (MDA) and nitrite levels. Before proceeding with the in vivo study, the Emorsan^®^ Gel was verified to be unabsorbed. Therefore, the observed effects could be ascribed to a local action. The results obtained in vivo showed that through local reduction of oxidative stress and inflammation (IL-1β, TNF-α), the Emorsan^®^ Gel significantly reduced the infiltration of mast cells into the injured wound, leading to the amelioration of symptoms such as itch and skin irritation. Therefore, the Emorsan^®^ Gel improved the speed and percentage of wound area closure by improving the tissue remodeling process, prompting vascular–endothelial growth factor (VEGF) and transforming growth factor (TGF)- β production and reducing the expression of adhesion molecules. Emorsan^®^ Gel, by its ability to inhibit free radicals, could reduce local inflammation and oxidative stress, thus enhancing the speed of wound healing.

## 1. Introduction

Skin wounding represents a common unfavorable event that may significantly affect patients’ quality of life [1]. The skin is the main organ of the integumentary system; it plays a primary role in the immune defense of the organism and in protecting against environmental stressors, such as chemicals, physical insults, and mechanical stimuli [2,3]. It performs multiple other functions, including mechanical, sensory, homeostatic, and endocrine functions [4].

Inadequate management of wounds, especially in chronic pathologies such as diabetes and immunocompromising pathologies, can lead to significant morbidity and contribute to increased mortality [2,3,5]. Successful wound care requires an in-depth understanding of skin regeneration mechanisms. Cutaneous wound healing typically develops through three phases: (1) an inflammatory stage, involving platelet aggregation and inflammatory mediator enrollment to the wound site; (2) a proliferative stage, characterized by the migration and proliferation of fibroblasts, keratinocytes, and endothelial cells, collectively leading to re-epithelialization and to the formation of granulation tissue; (3) and a protracted remodeling phase [6,7]. These processes repair tissues by hemostasis, inflammation, cell proliferation, and remodeling [8]. Therapeutic strategies in wound healing aim at restoring the vascular system, reducing inflammation, and promoting fibroblast differentiation, which will lead to the formation of connective tissues [9].

Recent scientific evidence [10,11] has highlighted the role played by oxidative stress (OS) in WH. The wounded tissue is characterized by an increased production of radical oxygen species (ROS) and nitrogen species (RNS), which can lead to cell membrane disorganization and protein oxidation, contributing to the impairment of the WH process [12]. Moreover, the persistent and uncontrolled production of ROS and RNS occurring during the inflammatory phase hinders physiological detoxification, further contributing to impaired WH. The antioxidative response, though elevated in chronic wounds, is unable to inhibit oxidants because of the imbalance between free radical species and antioxidant defense [10]; this results in an excessive increase in oxidant concentration. In chronic wounds, such imbalance is even more enhanced, especially during the inflammatory phase, further limiting the spontaneous production of granulation tissue that allows WH [13].

Given this scenario, effective oxidative stress management may become a valuable strategy to reduce tissue damage, improve WH, and limit the onset of chronic lesions [11].

Previous studies [14,15] have demonstrated the beneficial properties of different bacterial lysates for the management of several human health disorders due to their immunomodulatory capabilities. Among these, the lysate of *Corynebacterium granulosum* (now called *Propionibacterium acnes*) has shown promising results in combating vulvovaginal infections sustained by the impairment of the vaginal ecosystem [16]. Additionally, it alleviates symptoms in the management of atopic dermatitis. It was effective in treating irritant and allergic contact dermatitis triggered by soaps, solvents, chemicals, and cosmetics [17]. *Propionibacterium acnes*, present on the skin and constantly exposed to environmental stressors, is endowed with defense systems against OS, based on the scavenging action of the radical oxygenase of *Propionibacterium acnes* (RoxP), which has no homologs in other bacteria and is able to counteract OS by reducing free radicals [18]. Theoretically, if *Propionibacterium acnes* retains such antioxidative properties, the lysate would be an interesting candidate ingredient of topical formulations for managing WH. This study therefore aimed to assess in vitro some key properties of a gel containing a *Propionibacterium* extract (Emorsan^®^ Gel), including its antioxidant activity.In addition, the further aim of the study was to evaluate any other mechanism of action concerning the reepithelization effect in a mouse model of WH.

## 2. Materials and Methods

### 2.1. The Tested Formulation

Emorsan^®^ Gel (Depofarma S.p.A., Treviso, Italy) is a topical gel containing *Propionibacterium* extract, and it is supposed to act as an antioxidant agent. A previous experiment has shown the presence of *Propionibacteruim* extract in each sample made for clinical tests. The formulation contains dimethicone and cyclopentasiloxane, which exert a skin-protective action against external agents by forming occlusive barriers on the epidermis [19]; lactic acid, which helps in restoring the local pH to physiological levels [20]; and squalene, which protects the skin through the regeneration of the lipidic film and acts as a moisturizing agent as well.

### 2.2. Study Design

The study was designed to assess some key properties of Emorsan^®^ Gel in vitro and in vivo. First, the antioxidant activity was assessed using the ABTS and DPPH assays. Absorption of Emorsan^®^ Gel was evaluated using synthetic nitrocellulose membranes (Franz cell) and categorized according to the rate of device diffusion through the membranes over 24 h. Fibroblasts (L-929 cells) were used to evaluate the protection exerted by the bacterial extract against hydrogen peroxide (H_2_O_2_)-induced cytotoxicity and lipid peroxidation through a cell viability assay and malondialdehyde assay, respectively. Thereafter, the Emorsan^®^ Gel was evaluated for its ability to improve WH in a murine model. Briefly, given the role played by inflammation in slowing down WH, the effects of the gel on inflammatory factors, such as TNF-α and IL-1β, and tissue infiltration of mast cells were assessed [21]. Since inflammation is directly linked with symptoms, such as itch and skin irritation, scratching bouts and dermal irritation were also assessed. Further analyses included the evaluation of the expression of some factors involved in tissue regeneration, such as TGF-β [6], VEGF, ICAM-1, and p-selectin through immunohistochemical tests. Finally, global tissue repair was assessed by H/E staining.

### 2.3. Antioxidant Activity

The antioxidant fraction of the samples of the *Propionibacterium* extract, derived from three different production batches, was first extracted based on the method reported by Yu et al. [22]. Briefly, 1 g of the sample was added to 10 mL of absolute ethanol in nitrogen flow and stored in the dark for 15 h at room temperature. Thereafter, the mixture was centrifuged at 6000 rpm for 20 min, and the extract was stored at 4 °C until analysis.

The antioxidant activity was assessed following both the ABTS method, as described by Re et al. [23], and the DPPH method, as described by Yu et al. [22]. For both free radical scavenging assays, the reducing capacity of the *Propionibacterium extract* was measured with reference to the Trolox calibration curve (2–100 ppm) and expressed as a Trolox equivalent/kg sample.

### 2.4. Franz Cell Diffusion Study

A clean, dried receptor cell was filled with 25% ethyl and allowed to equilibrate at 32 ± 2 °C in the heated magnetic block for 30 min. The nitrocellulose membranes were trimmed into circular discs, mounted between the matched donor and receptor compartment. Emorsan^®^ Gel was placed on the membrane surface in the donor compartment. All openings, including donor top and receptor arm, were occluded with parafilm to prevent evaporation. The receptor compartment was stirred at 300 rpm. Using a glass syringe, sample volumes (1–2 mL) were extracted for UV assay (at 272 nm) and a fresh preheated replacement medium of the same volume was reintroduced into the receptor. Emorsan^®^ Gel was introduced into the donor at an approximate hourly interval to maintain its saturated state. The cumulative amount of Emorsan^®^ Gel cream diffusion over 24 h was recorded.

### 2.5. In Vitro Study

#### 2.5.1. Cell Culture

Murine fibroblast L-929 (NCTC clone 929 [L cell, L929, derivative of Strain L] ATCC^®^ CCL1 TM) were obtained from American Type Culture Collection (ATCC) and cultured in DMEM supplemented with 10% FBS, 100 units/mL penicillin, and 100 μg/mL streptomycin and maintained in a Thermo carbon dioxide incubator (Thermo Fisher Scientific, Waltham, MA, USA) at 37 °C with a humidified atmosphere of 95% air and 5% CO_2_.

#### 2.5.2. In Vitro Model Induction

Murine fibroblasts L-929 (4 × 10^4^ cells) were plated in 96-well plates, in a total volume of 150 μL for each well, and allowed to adhere for 4 h at 37 °C. Thereafter, the medium was replaced with a fresh one, and the cells were pre-treated with four different concentrations (0.01 μg/mL, 0.1 μg/mL, 1 μg/mL, and 10 μg/mL) of *Propionibacterium* extract (kindly provided by Depofarma S.p.A.) for 3 h and then stimulated with hydrogen peroxide (H_2_O_2_) for 10 min. To establish its effects on cell viability 3-(4,5-dimethylthiazol-2-yl)-2,5-diphenyltetrazolium bromide (MTT) colorimetric assay was performed. Supernatants were then subjected to a biochemical assay.

#### 2.5.3. Experimental Design

In summary, L-929 cell cultures were divided into 3 groups: (1) Control (CTR), cells were cultured in a normal culture medium; (2) CTR + H_2_O_2_, cells were stimulated with H_2_O_2_ 200 μM for 10 min; (3) H_2_O_2_ + *Propionibacterium* extract, cultures were pre-treated for 3 h with the *Propionibacterium* extract at different concentrations and then stimulated with H_2_O_2_ 200 μM for 10 min. The *Propionibacterium* extract was added with H_2_O_2_.

#### 2.5.4. Malondialdehyde (MDA) and Nitrite Evaluation by Biochemical Assay

Malondialdehyde (MDA) is one of the products of membrane lipid peroxidation; therefore, its levels are indicative of the degree of oxidative stress inflicted on biological membranes [24]. The extent of lipid peroxidation was evaluated using the TBARS assay kit, according to the manufacturer’s indications. Quantification of nitrite (NO_2_^−^) is an indicator of nitrosative stress, and it was performed using the Griess reagent, as previously described [25,26].

### 2.6. In Vivo Study

#### 2.6.1. Materials

Unless otherwise stated, all compounds were obtained from Sigma-Aldrich (St. Louis, MO, USA). All other chemicals were of the highest commercial grade available. All stock solutions were prepared in non-pyrogenic saline (0.9% NaCl, Baxter, Milan, Italy).

#### 2.6.2. Animals

Male adult CD1 mice (25–30 g; Envigo, Italy) were accommodated in a controlled habitat and provided with water and ordinary rodent food. Mice were located in stainless steel cages in a room kept at 22 ± 1 °C with a 12 h dark and 12 h light cycle. The animals were familiarized with their habitat, with free access to rodent standard diet and water ad libitum. Before being used in this study, the animals were kept in a quarantine area for one week. During this period, they were daily observed. At the end of the quarantine, the animals were carefully examined to evaluate their suitability for the study.

Messina University Review Board for the care of animals approved the study, and the approved project identification code was 294/2021-PR. All animal experiments were in accordance with the new Italian regulations (D.Lgs 2014/26) and EU regulations (EU Directive 2010/63). All experiments were conducted in accordance with ARRIVE guidelines.

#### 2.6.3. Wound Healing Induction Model

Wound healing was induced as previously described [6]. Mice were anesthetized with intraperitoneal xylazine and ketamine (0.16 and 2.6 mg/kg body weight, respectively). Hair on the back was clean-shaved by a depilatory cream, and the skin was slapped with a povidone–iodine solution and then cleaned with sterile water. Two full-thickness longitudinal cuts (4 cm) were made on the dorsum of the animals, and the wound boundaries were closed with surgical sutures (4-0 silk) at 1-cm intervals. Mice for each group were euthanized after 6 and 12 days, respectively, and the wounds were separated into two segments. The first segment was used for histology, and the second segment for molecular analysis.

#### 2.6.4. Experimental Groups

To evaluate the effects of Emorsan^®^ Gel in vivo, sham mice that did not undergo WH induction were compared to WH mice. Particularly, within the two groups, mice were randomly allocated to treatment with Emorsan^®^ Gel or vehicle for 6 or 12 days (N = 8 mice for each group, Table 1).

Emorsan^®^ Gel (200 mg/mouse) was administered topically starting 1 h after the WH procedure, then once daily for 6 and 12 days.

#### 2.6.5. Immunohistochemical Localization of TNF-α, IL-1β TGF-β, VEGF, ICAM-1, and P-Selectin

Immunohistochemical localization was performed, as previously reported by Campolo et al. [27].

Epidermal 5-µm slices were obtained after microtome cutting. Following the deparaffinization, endogenous peroxidase was quenched with 0.3% (*v*/*v*) H_2_O_2_ in 60% (*v*/*v*) methanol for 30 min. Slices were permeabilized with 0.1% (*w*/*v*) Triton X-100 in PBS for 20 min. Non-specific adsorption was decreased by incubating the section in 2% (*v*/*v*) normal goat serum in PBS for 20 min. Endogenous avidin and biotin binding sites were blocked by sequential incubation for 15 min with avidin and biotin (Vector Laboratories, Burlingame, CA, USA), respectively.

Subsequently, slices were incubated at room temperature overnight with one of the following primary antibodies: anti-transforming growth factor (*TGF)-β* (Santa Cruz Biotechnology #sc-17792, 1:100 in PBS, *v*/*v*), anti-intercellular adhesion molecule (ICAM)-1 (Santa Cruz Biotechnology #sc-8439, 1:100 in PBS, *v*/*v*), anti-P-selectin (Santa Cruz Biotechnology #sc-6941, 1:100 in PBS, *v*/*v*), anti-vascular endothelial growth factor (VEGF) (Santa Cruz Biotechnology #sc-7269, 1:100 in PBS, *v*/*v*), anti-tumor necrosis factor (TNF)-α (Santa Cruz Biotechnology #sc-52746, 1:100 in PBS, *v*/*v*), and anti-interleukin (IL)-1β (Santa Cruz Biotechnology #sc-32294, 1:100 in PBS, *v*/*v*). Thereafter, slices were washed with PBS and incubated with a secondary antibody (Santa Cruz Biotechnology, Dallas, TX, USA) for 1 h. The immunohistochemical reaction was revealed by a chromogenic substrate (brown DAB) counterstaining with nuclear fast red stain. To ascertain the binding specificity for different antibodies, some slices were incubated with the primary or secondary antibody only; no positive staining was observed in these sections. The percentage area of immunoreactivity, determined by the number of positive pixels, was expressed as a percentage of total tissue area (red staining). All stained sections were observed and analyzed in a blinded manner. For immunohistochemistry, images at 10× and 20× magnification (100 µm and 50 µm scale bar) were shown.

#### 2.6.6. Toluidine Blue Staining

Mast cell infiltration into epidermal tissues was evaluated by toluidine blue staining, as previously described [28]. The number of mast cells was evaluated on each slide using an Axiovision Zeiss microscope (Milan, Italy). For toluidine blue staining, images at 20× magnification (50 µm scale bar) and 40× magnification (20 µm scale bar) were shown.

#### 2.6.7. Pruritogen-Induced Scratching Behaviors

Itch represents an irritating cutaneous sensation that induces a scratch [29]. As reported by Upton et al. [30], in the wound healing process, patients report itching sensations within and around the wound site. This phenomenon is related to the regeneration of nerves in the growing wound tissue or skin’s physical interactions. Mice were placed in behavioral chambers on a Plexiglass platform and allowed to acclimate. Bouts of scratching were measured live in 5 min bins for 30 min following each treatment.

#### 2.6.8. Skin Irritation

Dermal irritation is an undesirable adverse event that may affect the wound healing area, thus concurring to the inflammatory reaction at the site of contact [31].

The general conditions of the animals were verified daily. Reactions were evaluated at different time points: 24, 48, and 72 h and 6, 10, and 12 days after exposure. Skin irritation was scored and recorded according to the scores reported in the following table (Table 2).

#### 2.6.9. Histopathological Analysis

Skin wound induction is characterized by a decrease in angiogenesis, delayed formation of granulation tissue, reduced collagen content, and lessened arteriolar quantity and density, together with a loss of vasculature tone and a decline in the cross-sectional area of novel vessel walls [6]. In relation to this, epidermal tissue integrity was analyzed by H/E staining to evaluate the morphological changes after WH induction. Wound specimens were fixed in 4% formaldehyde and embedded in paraffin. Sections were stained with hematoxylin and eosin (H&E) for histological analysis; the field was chosen to include all wound beds into a 2.5× and 10× optical field magnification. The histological score on a point scale of 0–4 was determined, as previously described. Briefly, the scores were 0, absence of epithelial proliferation in >70% of the tissue; 1, poor epidermal organization in >60% of the tissue; 2, incomplete epidermal organization in >40% of the tissue; 3, moderate epithelial proliferation in >60% of the tissue; and 4, complete epidermal remodeling in >80% of the tissue.

### 2.7. Malondialdehyde (MDA) Assay

The level of MDA, as an indicator of lipid peroxidation, is a useful marker of oxidative stress status. MDA assay in the wound tissues was determined as previously described by Scuderi et al. [32].

### 2.8. Nitrite/Nitrate Measurement

Nitrite/nitrate concentration were measured in the specimens using a Griess reaction assay kit as previously described [33] and expressed as mmol/mouse.

### 2.9. Statistical Analysis

All values were presented as mean ± standard error of the mean (SEM). The experiments were representative of at least three independent experiments performed on different days. Data were analyzed using one-way and two-way ANOVA, followed by a Bonferroni post-hoc test for multiple comparisons. Tests were considered significant if the *p*-value was <0.05.

## 3. Results

### 3.1. Antioxidant Activity of Bacterial Extract

The data obtained from DPPH and ABTS assays indicated that the bacterial extract was characterized by free radical (DPPH) and radical cation ABTS^+^ scavenging activities of 19.95 and 7.88 Trolox equivalent/kg *Propionibacterium* extract, respectively, as reported in Table 3.

### 3.2. Effect of Emorsan^®^ Gel Diffusion through Mice Epidermal Mucosa over 24 h

The diffusion test showed that, over the 24 h, Emorsan^®^ Gel was not detected in the receiving chamber of the Franz diffusion cell apparatus with synthetic nitrocellulose membranes (data not shown).

### 3.3. Effect of Propionibacterium Extract on Oxidative Stress

**Figure 1:**
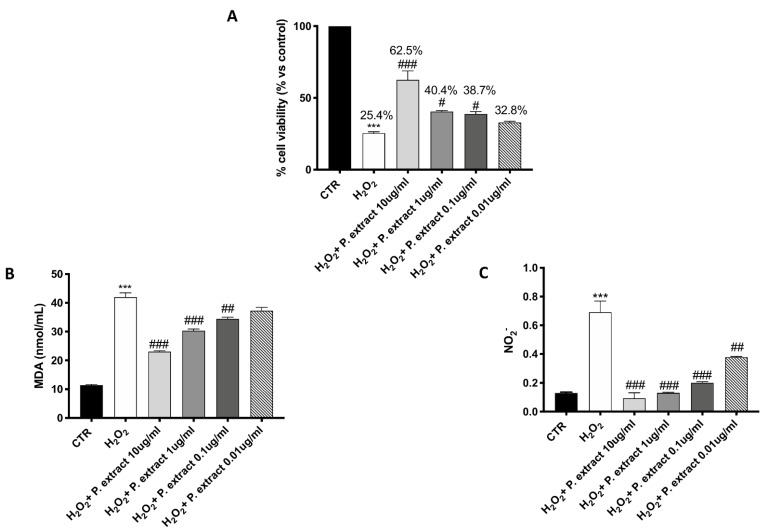
**Antioxidant effect of *Propionibacterium* extract in L-929 culture.** Pre-treatment with *Propionibacterium* extract, in a concentration-dependent manner, considerably prevented H_2_O_2_ cytotoxicity (**A**) and reduced MDA (**B**) and NO_2_^−^ (**C**) levels in the cell lysate, following H_2_O_2_ stimulation. Data are representative of at least three independent experiments. Values are provided as means ± SEM. One-way ANOVA test. *** *p* < 0.001 vs. CTR; # *p* < 0.05 vs. H_2_O_2_; ## *p* < 0.01 vs. H_2_O_2_; ### *p* < 0.001 vs. H_2_O_2_.

A significant protective effect against H_2_O_2_-induced oxidative stress was observed following *Propionibacterium* extract treatment, characterized by a concentration-dependent trend (Figure 1A). Treatment with the *Propionibacterium* extract notably prevented the elevated lipid peroxidation caused by H_2_O_2_ in a concentration-dependent manner (Figure 1B).

After the NO_2_^−^ assay, the untreated control group released low levels of NO_2_^−^, whereas H_2_O_2_ stimulation significantly increased NO_2_^−^ production (Figure 1C). All the concentrations of *Propionibacterium* extract significantly decreased NO_2_^−^ production (Figure 1C).

### 3.4. Effect of Emorsan^®^ Gel Administration on Proinflammatory Cytokines after Wound Induction

**Figure 2:**
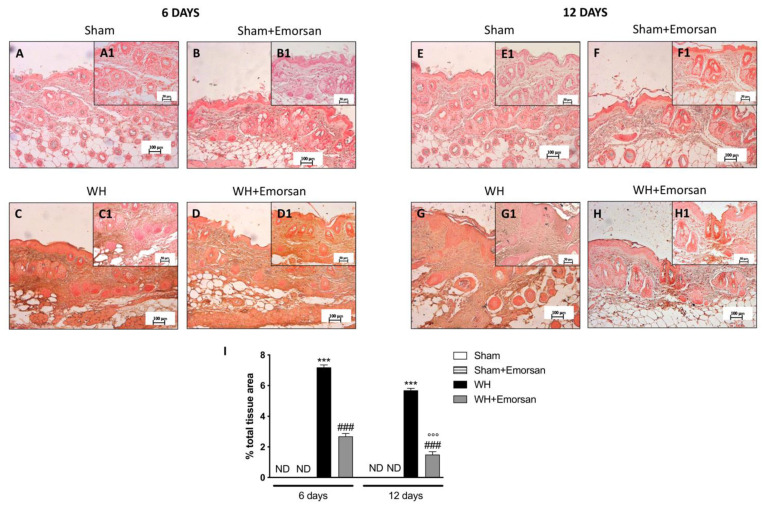
**Effect of Emorsan^®^ Gel treatment on TNF-α expression after WH.** Epidermal tissues collected from the WH-vehicle group revealed positive immunostaining for TNF-α (**C**,**C1**,**G**,**G1**,**I**), compared to the sham groups (**A**,**A1**,**B**,**B1**,**E**,**E1**,**F**,**F1**,**I**). Emorsan^®^ Gel topical treatment strongly reduced such positive immunostaining at both 6 days (**D**,**D1**,**I**) and 12 days (**H**,**H1**,**I**) after WH induction. Data are representative of at least three independent experiments. Values are means ± SEM. One-Way ANOVA test. *** *p* < 0.001 vs. corresponding Sham; ### *p* < 0.001 vs. corresponding WH; °°° *p* < 0.001 vs. WH+Emorsan 6 days; ND: not detectable.

Immunohistochemical analysis showed that TNF-α expression was absent in the sham groups (Figure 2A,A1,B,B1,E,E1,F,F1,I). Contrarily, TNF-α was significantly increased on day 6 (Figure 2C,C1,I) and day 12 (Figure 2G,G1,I) after wound induction. However, a substantial reduction in both cytokines was observed after the topical treatment of the wound with Emorsan^®^ Gel for 6 days (Figure 2D,D1,I) and 12 days (Figure 2H,H1,I).

**Figure 3:**
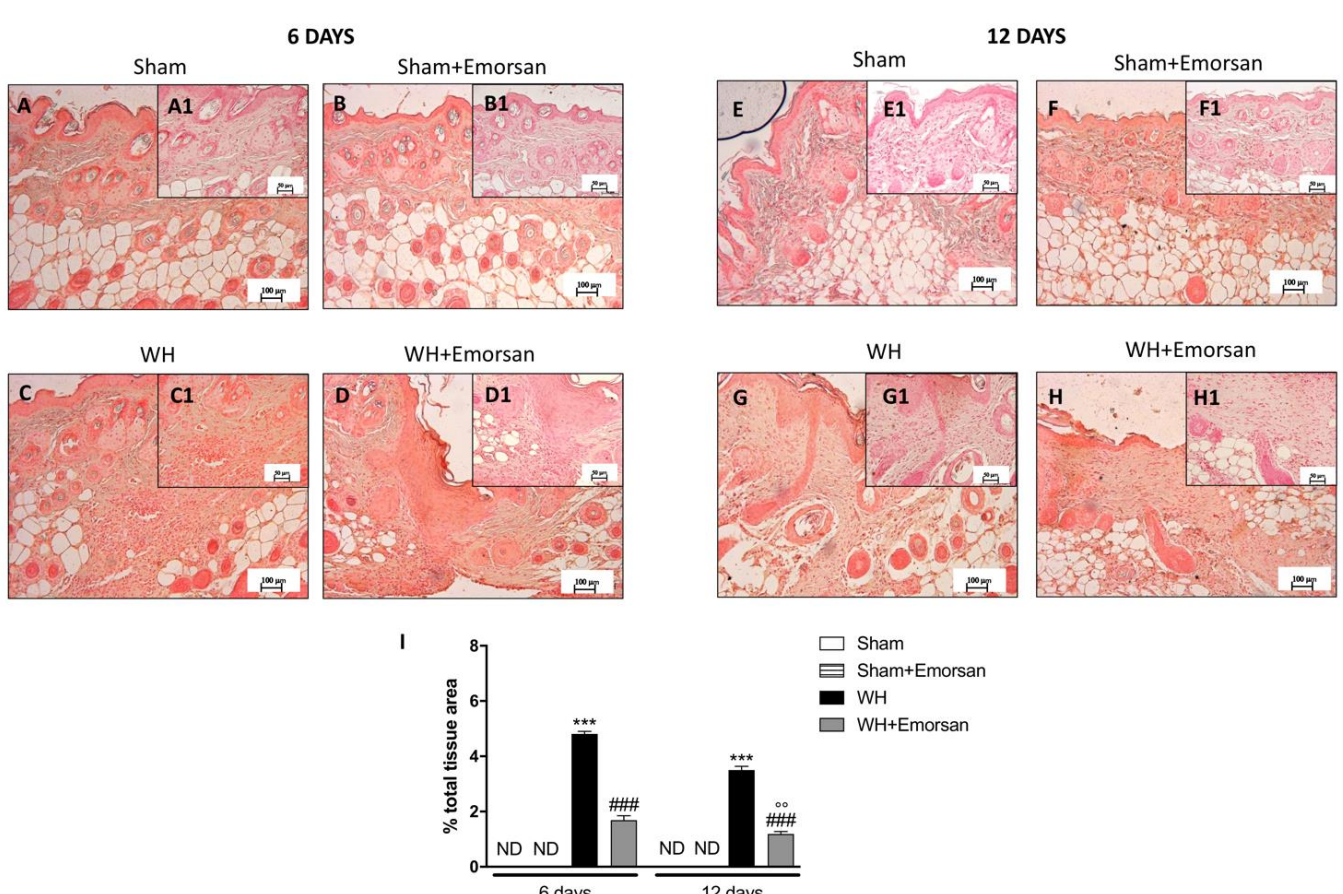
**Effect of Emorsan^®^ Gel treatment on IL-1β expression after WH**. Epidermal tissues collected from WH-vehicle injured mice showed positive IL-1β immunostaining (**C**,**C1**,**G**,**G1**,**I**), compared to the sham-treated mice 6 days and 12 days after WH induction (**A**,**A1**,**B**,**B1**,**E**,**E1**,**F**,**F1**,**I**). Emorsan^®^ topical administration reduced IL-1β expression in a significant way **(D**,**D1**,**H**,**H1**,**I**). Data are representative of at least three independent experiments. Values are means ± SEM. One-Way ANOVA test. *** *p* < 0.001 vs. corresponding Sham; ### *p* < 0.001 vs. corresponding WH; °° *p* < 0.01 vs. WH+Emorsan 6 days; ND: not detectable.

The evaluation of the wound tissues through immunohistochemical staining revealed the absence of IL-1β expression in the sham groups (Figure 3A,A1,B,B1,E,E1,F,F1,I). Differently, tissues from the WH+vehicle group presented an important overexpression of IL-1β both on day 6 (Figure 3C,C1,I) and day 12 (Figure 3G,G1,I) after wound induction. Topical treatment with Emorsan^®^ was able to decrease the levels of this cytokine after 6 days (Figure 3D,D1,I) and 12 days (Figure 3H,H1,I).

### 3.5. Effect of Emorsan^®^ Gel Administration on Mast Cells Infiltration after Wound Induction

**Figure 4:**
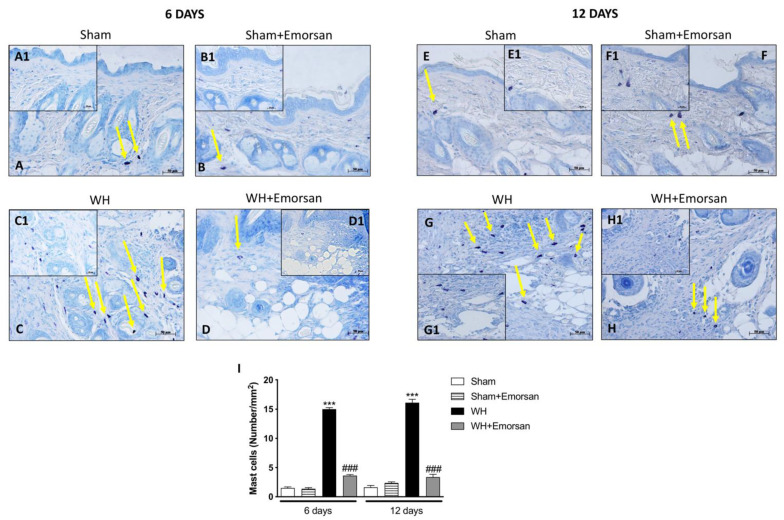
**Effect of Emorsan**^®^**Gel on mast cells in epidermal tissues after WH induction.** A high degree of mast cells was found in mice with WH (**C**,**C1**,**G**,**G1**,**I**) compared to control animals (**A**,**A1**,**B**,**B1**,**E**,**E1**,**F**,**F1**,**I**). However, a decreased number of mast cells was identified in mice topically treated with Emorsan^®^ at both 6 days (**D**,**D1**,**I**) and 12 days (**H**,**H1**,**I**). Data are representative of at least three independent experiments. Values are means ± SEM. One-Way ANOVA test. *** *p* < 0.001 vs. corresponding Sham; ### *p* < 0.001 vs. corresponding WH.

A minimal number of mast cells were found in the sham groups (Figure 4A,A1,B,B1,E,E1,F,F1,I). The analysis of mast cell infiltration, after the surgical incision procedure, showed a notable increase in the number of mast cells both on day 6 and day 12 (Figure 4C,C1,G,G1,I). Topical wound care with Emorsan^®^ was effective in considerably moderating mast cell infiltration in wound tissues (Figure 4D,D1,H,H1,score 4I).

### 3.6. Effect of Emorsan^®^ Gel Administration on Itch-Induced Scratching and Skin Irritation

**Figure 5:**
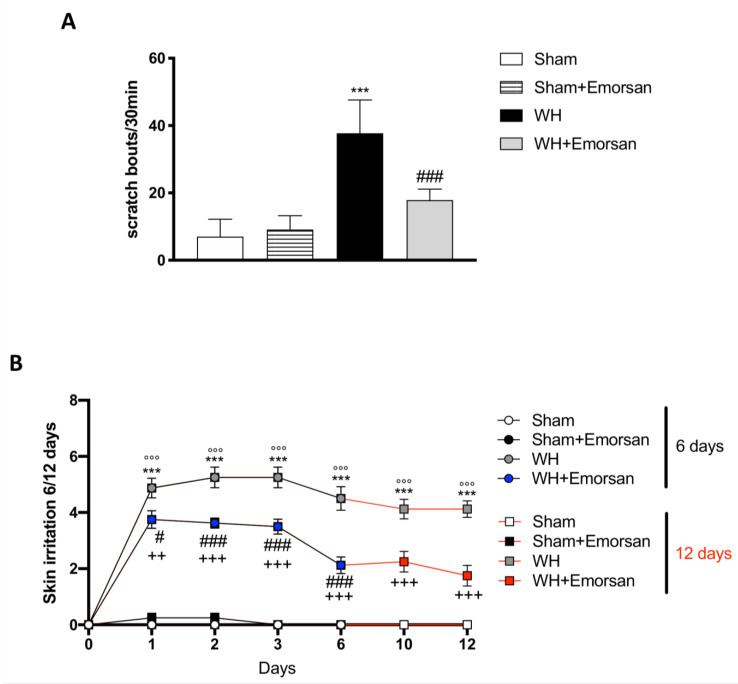
**Effect of Emorsan^®^ Gel administration on itch-induced scratching and skin irritation.** An increase in scratch bouts was detected in WH mice compared to sham animals (**A**); conversely, Emorsan^®^ Gel treatment was effective in reducing itch-induced scratching (**A**). Moreover, Emorsan^®^ Gel administration decreased skin irritation until 6 days and 12 days after WH induction (**B**). Data are representative of at least three independent experiments. Values are means ± SEM. One-Way ANOVA test. *** *p* < 0.001 vs. Sham 6 days; °°° *p* < 0.001 vs. Sham 12 days; # *p* < 0.05 vs. WH 6 days; ### *p* < 0.001 vs. WH 6 days; ++ *p* < 0.01 vs. WH 12 days; +++ *p* < 0.001 vs. WH 12 days.

Itch evaluation showed that Emorsan^®^ Gel is not irritating, as the total number of scratching bouts in 30 min in sham mice treated with Emorsan^®^ Gel was not statistically different from that observed in vehicle-treated sham mice. Contrarily, WH mice showed a significant increase in scratching bouts, compared to the sham group (Figure 5A), whereas Emorsan^®^-treated mice presented a considerable reduction in the total number of scratches (Figure 5A).

Furthermore, all sham groups displayed a very low skin irritation score at every time point (Figure 5B; row means ± SEM: 0.00 ± 0 for all sham groups at day 6 and day 12). Contrarily, already after 24 h from the wound induction, WH mice showed a considerable skin irritation score, which was even higher on day 6 (Figure 5B, row mean ± SEM: 4.50 ± 0.42) and day 12 (Figure 5B, row mean ± SEM: 4.13 ± 0.30). However, Emorsan^®^ treatment visibly reduced the skin irritation score, very appreciably on day 6 (Figure 5B, row mean ± SEM: 2.13 ± 0.30), and even more significantly on day 12 (Figure 5B, row mean ± SEM: 1.75 ± 0.37).

### 3.7. Effect of Emorsan^®^ Gel Administration on TGF-β Expression

**Figure 6:**
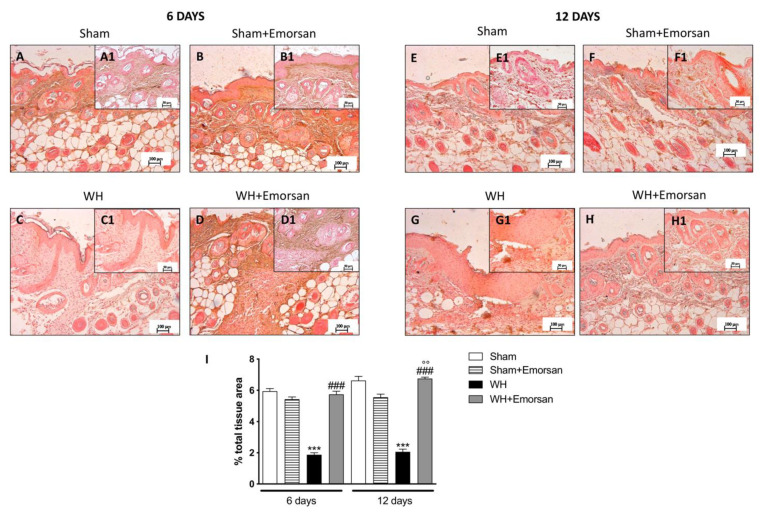
**Effects of Emorsan**^®^**Gel on TGF-β levels in the skin after 6 and 12 days from WH induction.** The epidermal tissues collected from WH-injured animals showed positive immunostaining for TGF-β (**C**,**C1**,**G**,**G1**,**I**), compared to the sham groups (**A**,**A1**,**B**,**B1**,**E**,**E1**,**F**,**F1**,**I**). Emorsan^®^ Gel topical administration notably reduced TGF-β levels in the skin after 6 and 12 days from WH induction (**D**,**D1**,**H**,**H1**,**I**). Data are representative of at least three independent experiments. Values are means ± SEM. One-Way ANOVA test. *** *p* < 0.001 vs. corresponding Sham; ### *p* < 0.001 vs. corresponding WH; °° *p* < 0.01 vs. WH+Emorsan 6 days.

Both on day 6 and day 12, high levels of TGF-β were identified in the sham groups (Figure 6A,A1,B,B1,E,E1,F,F1,I). Contrarily, WH-injured mice showed a reduction in TGF-β expression (Figure 6C,C1,G,G1,I), whereas the topical treatment with Emorsan^®^ Gel meaningfully increased TGF-β expression (Figure 6D,D1,H,H1,I).

### 3.8. Effect of Emorsan^®^ Gel Administration on VEGF Expression after Wound Induction

**Figure 7:**
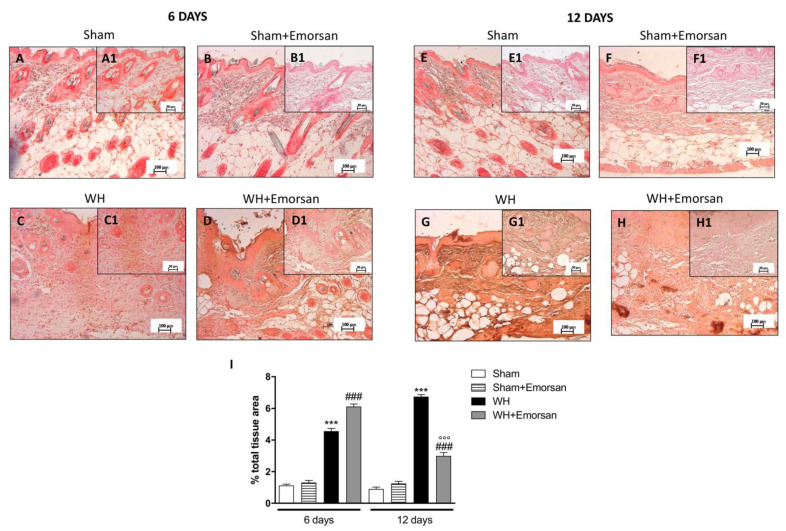
**Effect of Emorsan^®^ Gel treatment on VEGF expression after WH.** Mice of the vehicle group showed a marked positive immunostaining for VEGF after 6 days of WH induction (**C**,**C1**,**I**) compared to sham mice both on day 6 and day 12 after WH induction (**A**,**A1**, **B,B1,E,E1,F,F1,** **I**). In the vehicle group, such positive immunostaining was even more intense 12 days after WH induction (**G**,**G1**,**I**). Contrarily, Emorsan^®^-treated mice revealed a modulation of VEGF levels, which were increased 6 days after WH induction (**D**,**D1**,**I**), and decreased 12 days after the start of the experiment (**H**,**H1**,**I**). Data are representative of at least three independent experiments. Values are means ± SEM. One-Way ANOVA test. *** *p* < 0.001 vs. corresponding Sham; ### *p* < 0.001 vs. corresponding WH; °°° *p* < 0.001 vs. WH+Emorsan 6 days.

6 days after wounding an increase in VEGF-positive staining was found in the vehicle group (Figure 7C,C1,I), compared to the sham groups (Figure 7A,A1,B,B1,I). Topical treatment with Emorsan^®^ Gel was able to further increase VEGF staining (Figure 7D,D1,I). Instead, 12 days after wounding, VEGF levels were still increased in the vehicle group (Figure 7G,G1,I), compared to control animals (Figure 7E,E1,I), whereas VEGF expression significantly decreased after Emorsan^®^ treatment (Figure 7H,H1,I).

### 3.9. Effect of Emorsan^®^ Gel Administration on ICAM-1 and P-Selectin Expression after Wound Induction

**Figure 8:**
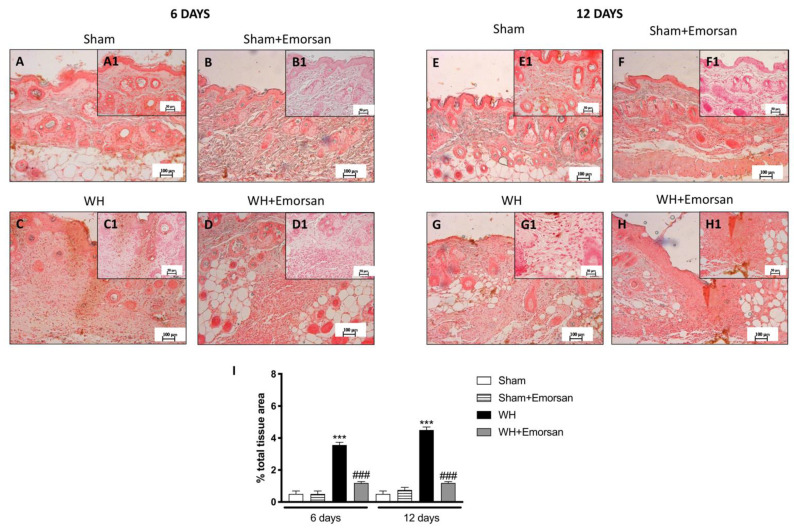
**Effect of Emorsan^®^ Gel treatment on ICAM-1 expression after WH.** Epidermal tissues obtained from WH-injured animals exhibited positive immunostaining for ICAM-1 (**C**,**C1**,**G**,**G1**,**I**), compared to the sham-treated animals (**A**,**A1**,**B**,**B1**,**E**,**E1**,**F**,**F1**,**I**). Emorsan^®^ Gel administered topically reduced this expression in both times (**D**,**D1**,**H**,**H1**,**I**). Data are representative of at least three independent experiments. Values are means ± SEM. One-Way ANOVA test. *** *p* < 0.001 vs. corresponding Sham; ### *p* < 0.001 vs. corresponding WH.

**Figure 9:**
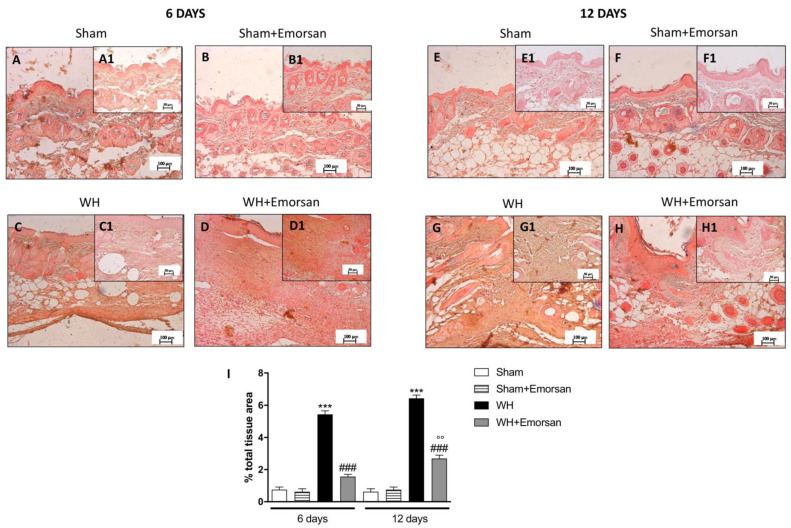
**Effect of Emorsan^®^ Gel treatment on P-Selectin expression after WH.** The intensity of P-selectin positive staining was markedly increased in the tissue section obtained from the WH group (**C**,**C1**,**G**,**G1**,**I**) compared to control animals (**A**,**A1**,**B**,**B1**,**E**,**E1**,**F**,**F1**,**I**). Mice topically treated with Emorsan^®^ Gel expressed a diminished expression of P-selectin positive staining (**D**,**D1**,**H**,**H1**,**I**). Data are representative of at least three independent experiments. Values are means ± SEM. One-Way ANOVA test. *** *p* < 0.001 vs. corresponding Sham; ### *p* < 0.001 vs. corresponding WH; °° *p* < 0.01 vs. WH+Emorsan 6 days.

Immunohistochemical analysis of adhesion molecules showed that on day 6 and day 12, from the beginning of the experiment, ICAM-1 and P-selectin expressions were low (Figure 8A,A1,B,B1,E,E1,F,F1,I and Figure 9A,A1,B,B1,E,E1,F,F1,I) in the sham groups. Otherwise, an increase in ICAM-1 and P-selectin positive staining was found in the WH-injured animals both on day 6 (Figure 8C,C1 and Figure 9C,C1, respectively, score Figure 9I) and day 12 (Figure 8G,G1 and Figure 9G,G1, respectively, score Figure 9I). Nevertheless, treatment with Emorsan^®^ decreased the levels of ICAM-1 and P-selectin, almost re-establishing them to the control levels 6 days (Figure 8D,D1 and Figure 9D,D1, respectively, score Figure 9I) and 12 days after damage (Figure 8H,H1 and Figure 9H,H1, respectively, score Figure 9I).

### 3.10. Effect of Emorsan^®^ Gel Administration on Epidermal Tissue Repair

**Figure 10:**
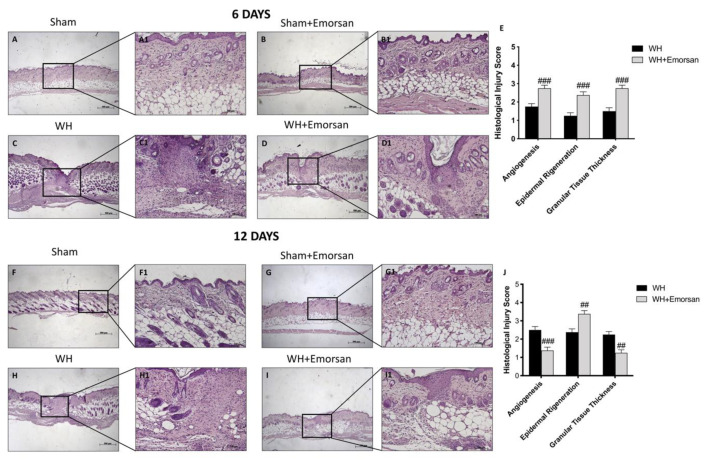
**Effect of Emorsan^®^ Gel treatment on histological parameters after WH induction.** WH-injured epidermis showed tissue damage, edema, and neutrophilic infiltration at both 6 days (**C**,**C1**,score **E**) and 12 days (**H**,**H1**,score **J**) compared to the sham mice (**A**,**A1**,score **E**,**F**,**F1**,score **J**). compared to the sham animals (**B**,(**B1**) histological score **G**,(**G1**), score **J**) at both 6 and 12 days. Emorsan^®^ Gel treatment significantly reduced tissue injury, ameliorating histolog-ical parameters (**D**,**D1**,score **E**,**I**,**I1**,score **J**). Data are representative of at least three independent experiments. Values are means ± SEM. One-Way ANOVA test. ## *p* < 0.01 vs. WH; ### *p* < 0.001 vs. WH.

H/E staining showed a substantially impaired wound characterized by a considerable neutrophilic infiltration, formation of edema, and restrained dermal and epidermal organization in WH-vehicle groups (Figure 10C,C1, histological score E,H,H1, histological score J), compared to the sham animals (Figure 10A,A1,B,B1 histological score E,F,F1,G,G1, histological score J) at both 6 and 12 days.

Emorsan^®^ Gel administration noticeably promoted re-epithelialization after 6 days and complete re-epithelialization after 12 days, thus exhibiting conventional differentiation and keratinization with epidermal elongation extending over two-thirds of the wound surface and acceptable glycogen storage to the margins of the wound area. Moreover, the granulation tissue appeared well structured, and dermal regeneration was characterized by granulation tissues rich in fibroblasts. In addition, treatment with Emorsan^®^ Gel led to a reduction in the number of inflammatory cells, which resulted in few polymorphonuclear cells scattered in the wound area and around the vessels (Figure 10D,D1, histological score E,I,(I1) histological score J).

### 3.11. Effect of Emorsan^®^ Gel Administration on Oxidative Stress Markers after Wound Induction

Considering the increased oxidative stress as a prominent player in regulating normal wound healing and to confirm in vitro experiment, we analyzed the effect of Emorsan^®^ administration on oxidative stress markers such as MDA and Nitrate/Nitrite levels.

**Figure 11:**
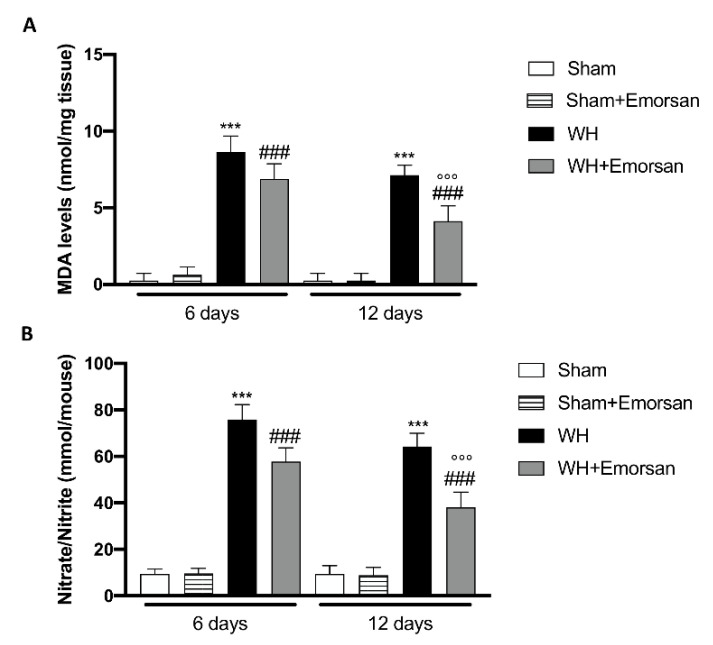
**Effect of Emorsan^®^ Gel administration on oxidative stress markers after wound induction**. Topical treatment with Emorsan^®^ Gel diminished MDA levels both on day 6 and day 12 after wound induction (**A**). Likewise, nitrate/nitrite levels were significantly decreased after Emorsan^®^ administration (**B**). Data are representative of at least three independent experiments. Values are means ± SEM. One-Way ANOVA test. *** *p* < 0.001 vs. corresponding Sham; ### *p* < 0.001 vs. corresponding WH; °°° *p* < 0.001 vs. WH+Emorsan 6 days.

The WH+vehicle group displayed a significant increase in MDA levels compared to the sham animals (Figure 11A), while treatment with Emorsan^®^ gel considerably reduced lipid peroxidation on day 6 and day 12 (Figure 11A). Moreover, we found that after wound induction, there was a significant increase in nitrate/nitrite levels on both day 6 and day 12 compared to the sham group (Figure 11B). Treatment with Emorsan^®^ gel protected the wound from oxidative stress, decreasing the production of free radicals at both times (Figure 11B).

## 4. Discussion

The *Propionibacterium* extract [18] exhibited relevant antioxidant properties. This was observed both in cell-free tests, such as ABTS and DPPH assays [34], and in cell cultures. The incubation of the bacterial extract with fibroblasts exerted a concentration-dependent reduction of MDA and nitrite levels, as well as a reduction in H_2_O_2_ cytotoxicity. Collectively, these results demonstrate, for the first time, the viability of the application of this bacterial extract to favor the protection of cells and tissues from OS. Considering the consequences of OS in lipid peroxidation and DNA or protein damage that may worsen the WH process by the induction of apoptosis or cellular senescence [35], these results highlight the potential of this bacterial extract to improve WH and limit unwanted skin consequences.

Considering possible topical applications of the extract, the permeation experiments proved that the effects are limited to the epidermal mucosa and are not attributable to a systemic absorption.

Results of in vitro tests are consistent with the anti-oxidative action observed in vivo, as OS characterizing skin wounds are linked to a direct inflammatory response. The response is supported by the infiltration of immune cells, such as mast cells [36,37,38], which in turn support inflammation and OS, establishing a vicious cycle that can ultimately delay re-epithelialization mechanisms [39].

In these regards, many scientific findings have elucidated the role of proinflammatory cytokines, and specifically of IL-1β and TNF-α, in orchestrating the manifold cellular activities underlying inflammation and healing [40,41]. The results obtained from this study clearly confirmed an extensive increase in pro-inflammatory cytokines, such as IL-1β and TNF-α, after wound injury induction at both 6 and 12 days. Contrarily, the topical administration of Emorsan^®^ was found to counteract the overexpression of both TNF- α and IL-1β, thus attenuating wound inflammation at 6 and 12 days after the injury. Moreover, a high degree of mast cell infiltration was observed 6 days after the wound procedure; the infiltration remained significant 12 days after the injury. Conversely, histological samples from wounds of Emorsan^®^-treated mice revealed a considerable decrease in the number of mast cells that were already visible from the sixth day. The results thus confirm that the local ROS scavenging effect promoted by Emorsan^®^ has beneficial consequences in wound tissue inflammation.

Such effects on inflammation markers are, in turn, consistent with those concerning the occurrence of symptoms associated with the impaired WH, such as acute or chronic itching [30]. Indeed, whereas a marked increase in scratch bouts and skin irritation was observed in WH-injured mice, topical administration of the Emorsan^®^ Gel effectively provided rapid symptom relief by decreasing both itching and WH-induced skin irritation at 6 and 12 days. If these results were observed in the clinical setting, they would be particularly relevant, because of the severe consequences of impaired wound healing on patients’ quality of life, compromising sleep quality and consequently their physical and psychological conditions [30,42]. Furthermore, scratching may lead to burns, infection recurrence, and delayed healing by exacerbating skin irritation [43].

Additionally, the results of the present study show the lysate as a relevant pro-reparative and a pro-regenerative agent. After WH injury, TGF-β expression was low in vehicle-treated mice both at 6 and 12 days after wound induction, whereas Emorsan^®^ administration resulted, at both time points considered, in a remarkable upregulation of TGF-β expression. This observation shows that the lysate has a pro-regenerative effect, as the TGF-β superfamily is a well-known mediator of tissue repair, and its modulation is known to possibly improve WH and scarring outcomes [44]. Specifically, the TGF-β1 isoform largely contributes to skin re-epithelialization by enhancing the migration of keratinocytes after binding to specific cell surface receptors [44].

Furthermore, TGF-β activity is known to strongly induce angiogenesis [45], specifically promoting VEGF expression at the skin injury site [6]. VEGF is released by a variety of cells that participate in wound repair [46], and its activity is substantially expressed through the stimulation of multiple components of the angiogenic cascade, resulting in an upregulation during the early days of healing, when capillary growth is maximal [46]. In this study, VEGF levels were observed to increase after treatment with Emorsan^®^ 6 days after injury, thus denoting positive outcomes in wound neoangiogenic processes. Otherwise, 12 days after WH induction, VEGF levels in Emorsan^®^-treated mice noticeably reduced, demonstrating that Emorsan^®^ Gel treatment was able to accelerate the healing process, leading to almost complete wound healing, whereby the intervention of VEGF was no longer required. Finally, an increased expression of ICAM-1 and P-selectin was observed in WH-untreated mice, but Emorsan^®^ Gel reduced this alteration both 6 and 12 days after wound induction. These adhesion molecules play a primary role in tissue repair and in cell migration, proliferation, and protein production [47]; their excessive activation is typically associated with skin inflammation. Their modulation could attenuate epidermal inflammatory reactions [48,49], as was observed following the application of the extract-containing gel.

The study revealed that the topical application of a gel containing *Propionibacterium* extract 6 and 12 days after wound induction was able to exert a local quenching effect against ROS and RNS, thus reducing the OS at the wound site. This led to a local reduction in inflammation and to the acceleration of the healing process after 6 days of treatment, ultimately ending in complete re-epithelialization after 12 days of treatment.

As impaired or defective WH may affect physiological and psychological aspects of functioning, new technics are sought to improve healing. Recently, nanocomposite hydrogels have been proposed to reduce time of healing thanks to their capability of providing structural support during the initial stages of tissue formation and their antibacterial properties [50,51].

The present study provides a new possible treatment option in the management of WH. Diverse categories of patients, including patients with diabetes and those undergoing surgical interventions, such as hemorrhoidectomy [52,53], could benefit from this possible treatment option. Therefore, the hypothesis that the oxidative scavenging properties of the bacterial extract-containing gel tested in the present study may be beneficial in favoring WH in the clinical setting may be tested by appropriate pilot clinical trials involving patients affected by these and other WH-impairing conditions.

## 5. Conclusions

Emorsan^®^ Gel, by its ability to inhibit free radicals, could reduce local inflammation and oxidative stress, thus enhancing the speed of wound healing in an in vivo model of WH. This finding could be applicable in the clinical setting, as a complementary tool for managing a wide set of conditions involving impaired WH. Further pilot clinical trials are needed to verify these findings and their clinical applicability.

## Figures and Tables

**Table 1:** Experimental groups.

Treatment	Days of Treatment	Number of Animals
SHAM		
Vehicle	6	8
Vehicle	12	8
Emorsan^®^ Gel	6	8
Emorsan^®^ Gel	12	8
WH		
Vehicle	6	8
Vehicle	12	8
Emorsan^®^ Gel	6	8
Emorsan^®^ Gel	12	8

**Table 2:** Scoring system for skin reaction.

Reaction	Irritation Score
***Erythema and eschar formation***	
No erythema	0
Very slight erythema (barely perceptible)	1
Well-defined erythema	2
Moderate erythema	3
Severe erythema (beet-redness)	4
***Oedema formation***	
No oedema	0
Very slight oedema (barely perceptible)	1
Well-defined oedema (edges of area well-defined by definite raising)	2
Moderate oedema (raised approximately 1 mm)	3
Severe oedema (raised more than 1 mm an extending beyond exposure area)	4
***Maximal possible score for irritation***	*8*

**Table 3:** Antioxidant evaluation through ABTS and DPPH assays.

Method	Trolox Equivalent/kg Bacterial Extract ± Standard Deviation
ABTS	19.95 ± 2.08
DPPH	7.88 ± 1.93

## Data Availability

The data presented in this study are available on request from the corresponding author.
